# Pan-cancer analysis reveals the prognostic and therapeutic relevance of MEX3A with a focus on chromophobe renal cell carcinoma

**DOI:** 10.3389/fonc.2026.1802679

**Published:** 2026-04-29

**Authors:** Shuang Wang, Jiejun Zhang, Mengyu Zheng, Yi Feng, Tao Li

**Affiliations:** 1 Nursing Department, The Fourth Affiliated Hospital of Harbin Medical University, Harbin, China; 2 Neurology Department, Yanda Hospital, Langfang, China; 3 Department of Cardiology, Kaifeng Central Hospital, Kaifeng, China; 4 Key Laboratory of Preservation of Human Genetic Resources and Disease Control in China (Harbin Medical University), Ministry of Education, Harbin, China; 5 Department of Cardiology, The Fourth Affiliated Hospital of Harbin Medical University, Harbin, China

**Keywords:** chRCC, MEX3A, drug sensitivity, immune regulation, genomic regulation, prognostic biomarker

## Abstract

**Background:**

MEX3A is an RNA-binding protein implicated in post-transcriptional regulation; however, its role across cancers, particularly in chromophobe renal cell carcinoma (chRCC), remains unclear.

**Methods:**

We performed a comprehensive pan-cancer analysis using TCGA and related databases to evaluate MEX3A expression, prognostic significance, immune associations, genomic alterations, and drug sensitivity. Functional experiments were conducted in chRCC cell lines.

**Results:**

MEX3A was significantly upregulated in multiple cancers and was associated with poor prognosis. Its expression correlated with immune cell infiltration, immune checkpoint molecules, tumor mutational burden, and microsatellite instability. High MEX3A expression was linked to reduced drug sensitivity. Functional assays confirmed its oncogenic role in chRCC.

**Conclusion:**

MEX3A serves as a potential prognostic biomarker and therapeutic target in chRCC and may play a critical role in tumor progression and immune regulation.

## 1 Introduction

Cancer continues to be a major cause of human mortality globally. Projections indicate that by 2040, there will be approximately 29.9 million cancer cases and 15.3 million deaths, underscoring the urgency to strengthen cancer care systems (1). Importantly, it is not a uniform disease but a heterogeneous ensemble of over 100 distinct malignancies, each with unique clinical features, genetic architectures, and therapeutic sensitivities (2). This complexity demands innovative strategies to decode its underlying mechanisms. Historically, cancers have been classified by tissue of origin, a framework that has long guided diagnosis and treatment. However, this approach fails to capture the molecular heterogeneity within and across types. For example, breast cancer (BRCA)-the most common malignancy in women-includes subtypes with divergent mutations, hormone receptor profiles, and outcomes (3). Similarly, lung cancer, the top cause of cancer deaths, divides in Non-Small Cell Lung Cancer (NSCLC) and SCLC(Small Cell Lung Cancer) types, with NSCLC further subclassified into adenocarcinoma and squamous cell carcinoma, each with distinct genetic alterations and treatment vulnerabilities (4). Despite such diversity, accumulating evidence reveals conserved molecular drivers of tumorigenesis: dysregulated cell cycle control, apoptosis evasion, enhanced angiogenesis, and metastatic activation. These shared hallmarks have spurred pan-cancer research, which seeks common molecular signatures, pathways, and therapeutic targets across malignancies (5). This approach transcends tissue boundaries, enabling broad-applicability therapies. High-throughput technologies Next-Generation Sequencing (NGS), microarrays, and mass spectrometry-based proteomics-have revolutionized pan-cancer studies. Large-scale projects like TCGA, profiling over 11, 000 tumors across 33 types, have generated vast multi-omics datasets (6). These resources uncovered recurrent mutations (Tumor Protein P53 (TP53), Kirsten Rat Sarcoma Viral Oncogene Homolog (KRAS)), copy number alterations (CNA), and shared expression patterns, deepening understanding of core oncogenic processes (7, 8). Key pan-cancer insights include identifying universally dysregulated “driver” genes and pathways. TP53 mutations, present in 50% of tumors, disrupt tumor suppression, while PI3K-AKT-mTOR pathway dysregulation recurrently drives cell growth and survival across cancers (9, 10). Such discoveries are critical for cross-cancer targeted therapies. Pan-cancer research has also accelerated biomarker identification: Epidermal Growth Factor Receptor (EGFR) mutations in NSCLC and BRCA mutations in breast or ovarian cancer guide therapy, while prostate and colorectal aid monitoring (11, 12). chRCC accounts for approximately 5% of renal cell carcinoma cases and exhibits distinct clinical and pathological features. Although chRCC generally follows an indolent course, it tends to exhibit local invasion and distant metastasis, and patients with advanced disease show limited responses to targeted and immunotherapeutic interventions.

Among emerging oncogenic regulators, MEX3A attracts attention. As an RNA-binding protein in the MEX3 family, it harbors KH domains (RNA binding) and a RING finger domain (E3 ubiquitin ligase activity), enabling post-transcriptional and post-translational gene regulation (13). Initial studies link MEX3A to stem cell self-renewal, differentiation, and apoptosis. In cancer, it is often upregulated: in colorectal cancer (CRC), high MEX3A correlates with advanced stage, metastasis, and poor prognosis, promoting invasion and Epithelial-Mesenchymal Transition (EMT) via Wnt/β-catenin activation, in clear cell renal carcinoma, ETS Proto-Oncogene 1(ETS1)-transactivated MEX3A regulates cell cycle, with silencing inducing G1/S arrest, in Acute Myeloid Leukemia (AML), its overexpression and knockdown-induced apoptosis suggest therapeutic potential (14, 15). Chromophobe renal cell carcinoma (chRCC) is a rare but distinct subtype of renal cell carcinoma, accounting for approximately 5% of all renal malignancies. Although chRCC generally shows an indolent clinical course in the early stage, patients with metastatic or recurrent disease face extremely poor prognosis due to limited effectiveness of targeted therapy and immunotherapy. The lack of effective prognostic biomarkers and therapeutic targets has become a major clinical dilemma for chRCC. Accumulating evidence has confirmed that MEX3A functions as an important oncogene in multiple tumors by regulating cell proliferation, metastasis, and drug resistance. Based on its critical role in tumor progression, we hypothesized that MEX3A may also participate in the malignant progression of chRCC and serve as a potential biomarker (15). However, MEX3A’s pan-cancer role in chRCC remains unclear. Critical knowledge gaps include the consistency or context-dependence of its expression across cancers, the genetic and epigenetic alterations driving its dysregulation, the variability of its functional roles (tumorigenesis, metastasis) across cancer types, and its potential as a pan-cancer biomarker or therapeutic target. Addressing these gaps requires comprehensive pan-cancer analysis, integrating genomic, transcriptomic, proteomic, and clinical data to characterize MEX3A’s expression, regulation, and function across malignancies. This investigation would clarify its conserved and context-specific roles, bridging gaps in cross-cancer regulatory networks.

In summary, while cancer remains a global challenge, pan-cancer research offers universal insights. MEX3A’s emerging role in diverse cancers underscores the need for systematic pan-cancer analysis. Elucidating its mechanisms across malignancies could identify novel biomarkers and targets, benefiting patients across cancer types. This study systematically analyzed MEX3A across cancers and highlights its potential as a prognostic and therapeutic target in chRCC.

## 2 Materials and methods

### 2.1 Expression, survival prognosis analysis and mutation

HPA database (https://www.proteinatlas.org/) is a database based on proteomics, transcriptomics, and systems biology data to map tissues, cells, and organs. We characterized the overall expression level of MEX3A through the HPA database (16). Furthermore, the expression of MEX3A in different cell lines was obtained through the CCLE database (https://registry.opendata.aws/ccle). RNA sequencing and clinical information for 33 cancers were obtained from the TCGA database (https://portal.gdc.cancer.gov/). DNA copy number data were downloaded from the CBIOPORTAL database (cBioPortal for Cancer Genomics) (17).

MEX3A expression was assessed in 33 tumors using downloaded data and expression levels were compared between cancer samples and matched standard samples in 33 cancers. Expression data were log2 transformed and two-group t-tests were performed on these tumor types. *p* < 0.05 was considered to indicate differences in expression between tumor and normal tissue. Box plots were drawn using the R software (version 4.3.1) “ggpubr” package.

To examine the relationship between MEX3A expression and patient prognosis, univariate Cox regression analysis of MEX3A for OS, DSS, DFI, and PFI was performed using the Sangerbox tool http://sangerbox.com/ (18). The cBio Cancer Genomics Portal (http://cbioportal.org) (19) is an open platform for interactive research of all-round cancer genomics datasets in the context of clinical data and biological pathways. We used cBioPortal to analyze MEX3A mutation level and mutation diagram of MEX3A in different cancer types across protein domains in the TCGA pan-cancer database.

### 2.2 The association between MEX3A expression and different tumor immune and molecular subtypes

TISIDB is a database that combines various data types to study the interaction between tumors and the immunology (cis.hku.hk/TISIDB/) (20). It was utilized to investigate the relationship between MEX3A expression and the immune or molecular subtypes of various cancers. Different immune types are summarized as follows: C1 (wound healing); C2 (IFN-gamma dominant); C3 (inflammatory); C4 (lymphocyte depleted); C5 (immunologically quiet); C6 (TGF-b dominant).

### 2.3 Correlation analysis between MEX3A and immune checkpoints, immune cells, immune inhibitors, immune scores, immune stimulators, and receptors

Correlation between the expression of MEX3A and the expression of immune checkpoint-associated genes in pan-cancer was analyzed by Pearson’s analysis of data from TCGA database. The immune cell infiltration score for pan-cancer was calculated by the CIBERSORT algorithm, and the correlation between MEX3A expression and 22 immune cell scores was analyzed by Pearson. Correlation between MEX3A expression and expression of immunosuppressant-related genes in pan-cancer was analyzed by Pearson.

### 2.4 Immunological differential analysis of MEX3A and immune microenvironment related scoring analysis and the correlation analysis of MEX3A with TMB, MSI

For immune infiltration analysis, CIBERSORT was employed to quantify the proportions of 22 immune cell subsets from gene expression profiles. Gene expression data and MEX3A expression information were obtained from relevant databases. Samples were divided into MEX3A high and low expression groups. R packages like including ggplot2 and maftools were used for data processing and visualization, generating stacked bar plots to display immune cell proportion differences. Statistical tests were performed to assess significance. To analyze the immune microenvironment associated with MEX3A, we first obtained gene expression profiles and corresponding clinical data from public databases. We used the “maftools” package in R software to organize the SNV data downloaded from the TCGA database in multiple alignment format. We also assessed tumor mutational burden (TMB) for each sample. microsatellite instability (MSI) refers to the nucleotide insertions or deletions in the microsatellite loci. Spearman’s rank method was used to determine the correlation of MEX3A with TMB and MSI. The correlation results for TMB and MSI were visualized in radar maps.

### 2.5 Correlation analysis of MEX3A with related genes in chRCC and mutation analysis of MEX3A in chRCC

For gene expression correlation heatmaps in chRCC, the pheatmap package in R was used to visualize correlations, with color scales indicating expression levels. Mutation landscape plots were generated via the maftools package, showcasing mutation types and frequencies in MEX3A low and high-expression groups. Statistical analyses and plot customizations were performed using R functions to ensure clarity for scientific publication.

### 2.6 In chRCC, the analysis of drug sensitivity and the correlation with drug sensitivity for MEX3A

Drug sensitivity was assessed using the R package oncoPredict based on gene expression profiles. Predicted half-maximal inhibitory concentration (IC50) values were calculated for each sample, where lower IC50 values indicate higher drug sensitivity, whereas higher IC50 values reflect reduced sensitivity. ChRCC samples were stratified into high- and low-expression groups according to the median MEX3A expression level, and differences in predicted IC50 values between the two groups were evaluated using the Wilcoxon rank-sum test. Drug sensitivity data and corresponding MEX3A expression profiles were obtained from TCGA database. Correlation analyses between MEX3A expression and predicted IC50 values were performed using the cor.test function in R. Scatter plots with fitted regression lines and 95% confidence intervals were generated using the ggplot2 package, with correlation coefficients (R) and P values calculated and displayed for each drug.

### 2.7 Cell culture and transfection

The renal cell carcinoma cell lines OS-RC-2 and A498 were purchased from Shanghai Zhongqiaoxinzhou Biotechnology Co., Ltd. (Shanghai, China). Cells were cultured in RPMI-1640 medium (KeyGEN BiOTECH, China) supplemented with 10% fetal bovine serum (Cell Box, China) and 1% penicillin-streptomycin at 37 °C in a humidified atmosphere containing 5% CO_2_. Cells were passaged every 2 days using 0.25% trypsin-EDTA (Solarbio, China) when reaching 80-90% confluence, and only cells from passages 3–8 were used for experiments to ensure consistent biological characteristics.

Small interfering RNAs (siRNAs) targeting MEX3A and non-targeting negative control siRNA (si-NC) were designed and synthesized by (Sangon Biotech, China). The sequences of si-MEX3A-1, si-MEX3A-2, and si-NC are listed in **Supplementary Table 1**. For transfection, OS-RC-2 cells were seeded into 6-well plates (2 × 10^5^ cells/well) and cultured for 24 h until 50–60% confluence. Transfection was performed using RNAtransMate (Sangon Biotech, China) following the manufacturer’s protocol. At 48 h post-transfection, cells were collected for subsequent experiments to verify transfection efficiency and assess biological effects.

### 2.8 Western blot

Total protein was extracted using RIPA lysis buffer supplemented with protease inhibitors. Protein concentration was determined using a BCA assay. Equal amounts of protein were separated by SDS-PAGE and transferred onto PVDF membranes. The membranes were blocked with 5% non-fat milk for 1 h at room temperature and incubated overnight at 4 °C with primary antibodies. Primary antibodies used were as follows: anti-MEX3A (rabbit polyclonal, Abmart, Shanghai, China; dilution 1:1000), anti-BCL2 (rabbit monoclonal, Abmart; 1:1000), anti-BAX (rabbit monoclonal, Abmart; 1:1000), and anti-GAPDH (mouse monoclonal, ZSGB-BIO, Beijing, China; catalog no. TA-08; 1:5000). After washing, membranes were incubated with HRP-conjugated secondary antibodies for 1 h. Protein bands were visualized using enhanced chemiluminescence (ECL) reagents.

### 2.9 CCK-8 assay

Cell proliferation was evaluated using the Cell Counting Kit-8. Briefly, cells transfected with si-NC, si-MEX3A-1, or si-MEX3A-2 were seeded into 96-well plates at an equal density. At indicated time points (0, 1, 2, and 3 days), 10 μL of CCK-8 solution was added to each well and incubated for 2 h at 37 °C. The absorbance was measured at 450 nm using a microplate reader. Cell viability was expressed as absorbance values.

### 2.10 EdU assay

Cell proliferation was assessed using an EdU Cell Proliferation Kit (Vazyme, China). Briefly, transfected cells were incubated with EdU reagent for 2 h at 37 °C, fixed with 4% paraformaldehyde, and permeabilized with 0.5% Triton X-100. Cells were then subjected to EdU staining according to the manufacturer’s instructions, followed by nuclear counterstaining with DAPI. EdU-positive cells were observed under a fluorescence microscope and quantified as a percentage of total cells. Experiments were performed in triplicate.

### 2.11 Wound healing assay

Cell migration was assessed using a wound healing assay. Briefly, cells transfected with si-NC, si-MEX3A-1, or si-MEX3A-2 were seeded into six-well plates and grown to approximately 90% confluence. A linear wound was generated using a sterile 200-μL pipette tip, and detached cells were gently removed by washing with PBS. Cells were subsequently cultured in serum-free medium. Images of the wound area were captured at 0, 12, and 24 h under an inverted microscope. Migration distance was measured using ImageJ software. All experiments were performed independently in triplicate.

### 2.12 Statistical analysis

All data for gene expression were standardized by log2 transformation. Independent t-test was used for comparison of normal and cancerous tissues. *p* < 0.05 indicated statistical significance. In this study, Kaplan-Meier curve, log-Rank test and COX regression model were used. Correlation analysis between two variables was performed using Spearman or Pearson tests; *p* < 0.05 was considered significant. All statistical analyses were processed by R software (version 4.3.1).

## 3 Results

### 3.1 Baseline expression characteristics of MEX3A in normal tissues and cancer cell lines

To clarify the physiological expression pattern of MEX3A, its transcriptional and protein expression profiles were systematically examined using publicly available datasets (**Supplementary Figure S1**). Analysis of the Human Protein Atlas demonstrated that MEX3A exhibited pronounced tissue specificity at the RNA level, with relatively high expression observed in the testis, brain, and selected digestive tissues, whereas most somatic tissues showed low or undetectable expression (**Supplementary Figure S1A**). Protein expression displayed heterogeneous distribution among tissues, implying potential post-transcriptional regulation (**Supplementary Figure S1B**). Consistent with these findings, GTEx data confirmed enrichment of MEX3A transcripts in restricted tissue types(**Supplementary Figure S1C**). Furthermore, evaluation of the CCLE dataset revealed marked variability of MEX3A expression across cancer cell lines, suggesting lineage-dependent dysregulation of MEX3A during tumorigenesis(**Supplementary Figure S1D**).

### 3.2 Pan-cancer analysis of MEX3A expression differences and genomic alterations between tumor and normal tissues features

**Figure 1:**
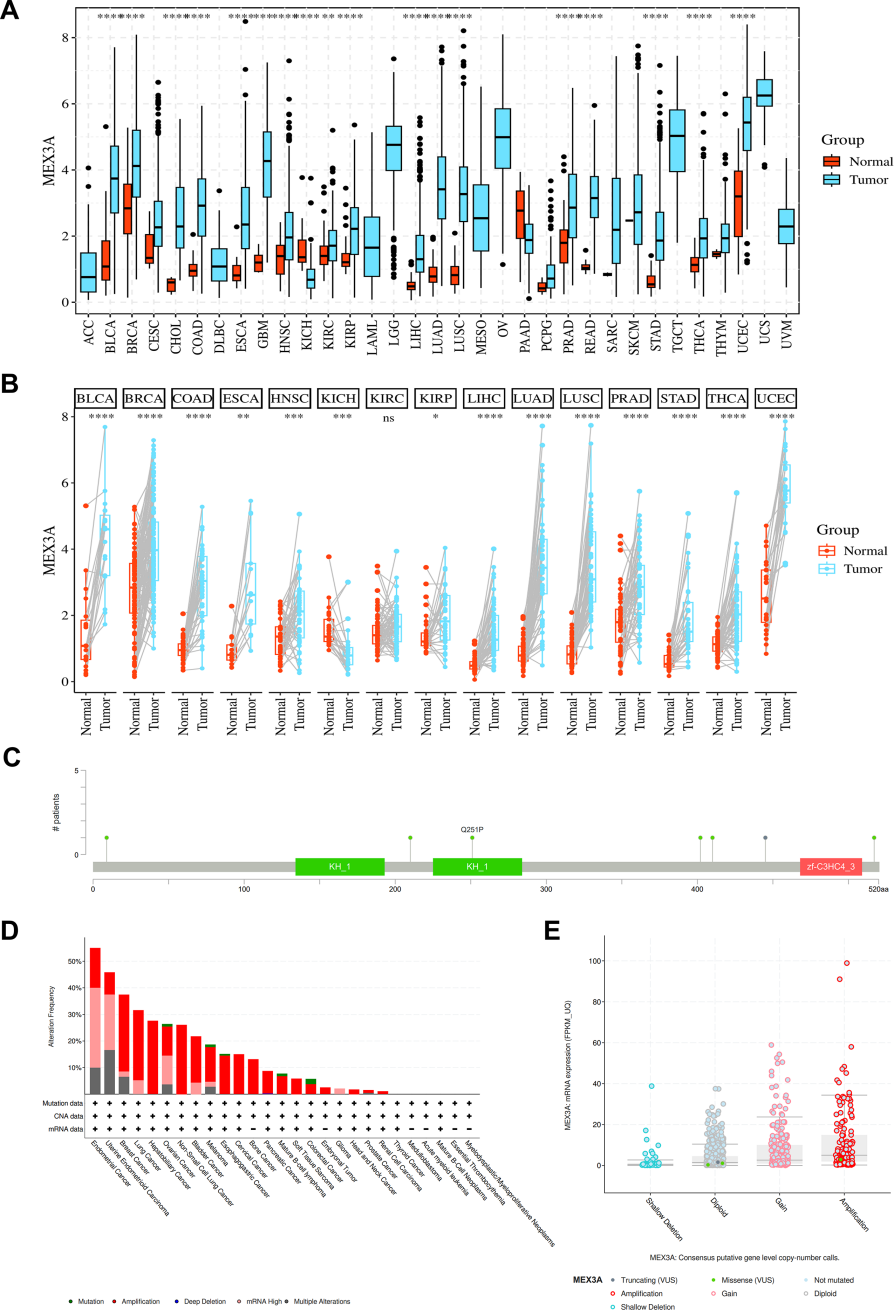
Pan-cancer analysis of MEX3A expression and genomic alterations. **(A)** MEX3A expression in tumor and normal tissues. **(B)** Paired tumor-normal expression. **(C)** Mutation map of MEX3A. **(D)** Genomic alterations of MEX3A. **(E)** Copy number alterations. *p<0.05, **p<0.01, ***p<0.001, ****p<0.0001.

To investigate whether MEX3A expression is altered in malignancies, pan-cancer analyses were conducted using TCGA datasets (**Figure 1**). Comparative analyses between tumor tissues and corresponding normal controls revealed that MEX3A expression was significantly elevated in a broad spectrum of cancers (**Figure 1A**). Paired analyses of matched tumor and adjacent normal tissues further confirmed consistent upregulation of MEX3A in tumors (**Figure 1B**). Genomic alteration profiling revealed that MEX3A harbored a relatively low mutation frequency across cancers. Copy number analysis indicated that gene amplification was the predominant alteration type affecting MEX3A in multiple tumor entities. Mapping of mutation sites across the MEX3A protein structure did not identify clear hotspot regions, suggesting that changes in gene dosage rather than recurrent point mutations may be the primary driver of MEX3A dysregulation in cancer. These results indicate that aberrant overexpression of MEX3A represents a common molecular feature across multiple cancer types (**Figures 1C–E**).

### 3.3 Prognostic significance of MEX3A across cancers

**Figure 2:**
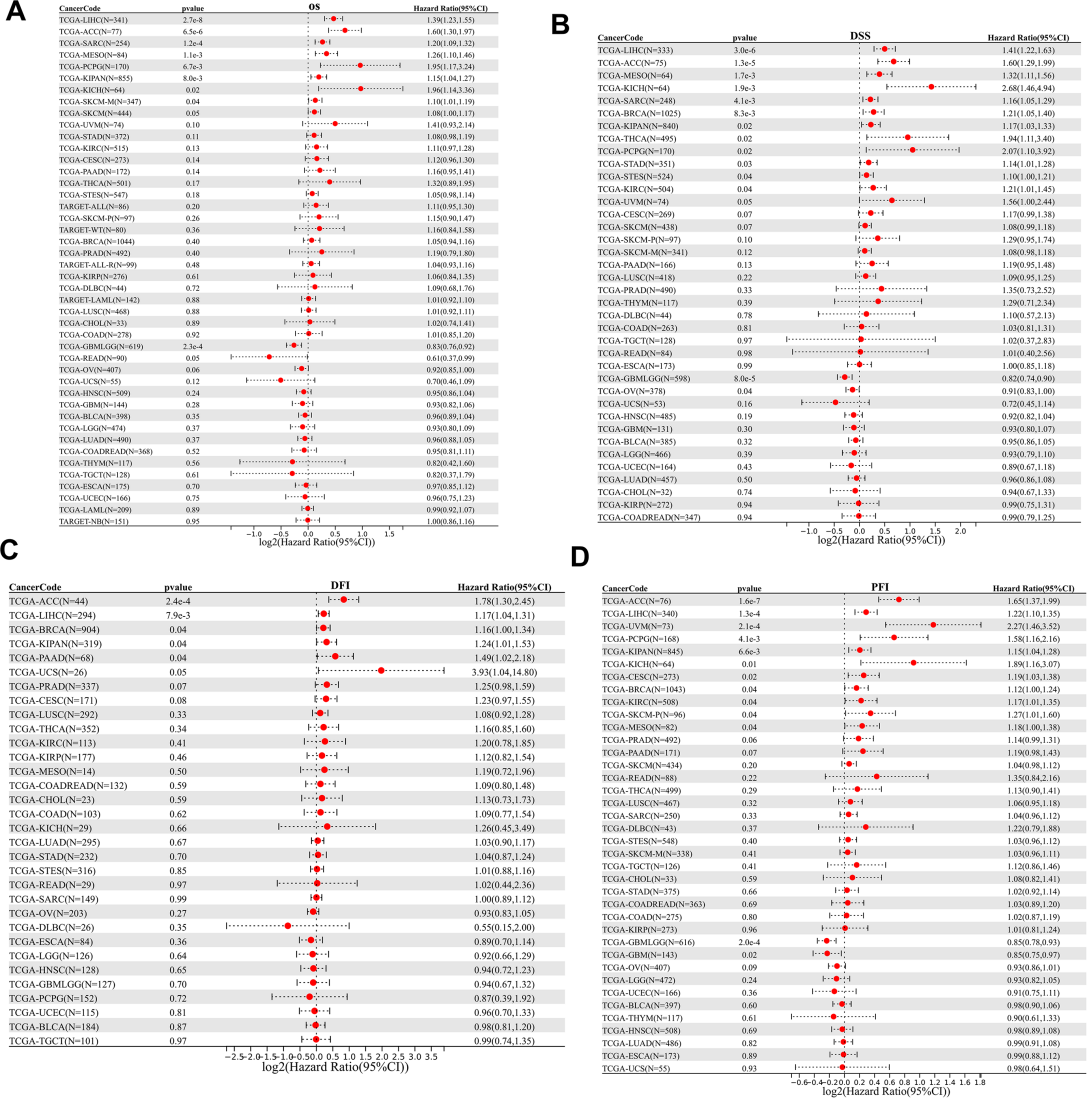
Pan-cancer prognostic and pan-cancer mutation landscape of the MEX3 impact of MEX3A in chRCC. Forest plots show the association between MEX3A expression and overall survival **(**OS, **A)**, disease-specific survival **(**DSS, **B)**, disease-free interval **(**DFI, **C)**, and progression-free interval **(**PFI, **D)** across pan-cancer cohorts. *p < 0.05, **p < 0.01, ***p < 0.001.

Across pan-cancer cohorts, the prognostic significance of MEX3A was systematically evaluated using univariate Cox regression analyses for overall survival (OS), disease-specific survival (DSS), disease-free interval (DFI), and progression-free interval (PFI) (**Figures 2A–D**). As illustrated in the forest plots, MEX3A expression exhibited significant associations with patient outcomes in multiple cancer types, although the direction and magnitude of these effects varied substantially across malignancies.

For OS and DSS, elevated MEX3A expression was predominantly associated with increased mortality risk in several cancers, including adrenocortical carcinoma (ACC), Liver hepatocellular carcinoma (LIHC), Sarcoma (SARC), Mesothelioma (MESO), and chRCC. Similarly, analyses of DFI and PFI revealed that higher MEX3A levels correlated with an increased risk of tumor recurrence or disease progression in selected cancer types. In contrast, weak, nonsignificant, or inverse associations were observed in other malignancies, highlighting the heterogeneity of MEX3A-related prognostic effects. Collectively, these findings demonstrate that MEX3A serves as a context-dependent prognostic factor across cancers, exerting tumor-specific influences on survival and disease progression rather than acting as a universal prognostic biomarker. Among these cancer types, chRCC showed a significant and consistent association between MEX3A and prognosis; therefore, we focused on chRCC in subsequent analyses.

### 3.4 Association of MEX3A with immune and molecular subtypes of tumors

**Figure 3:**
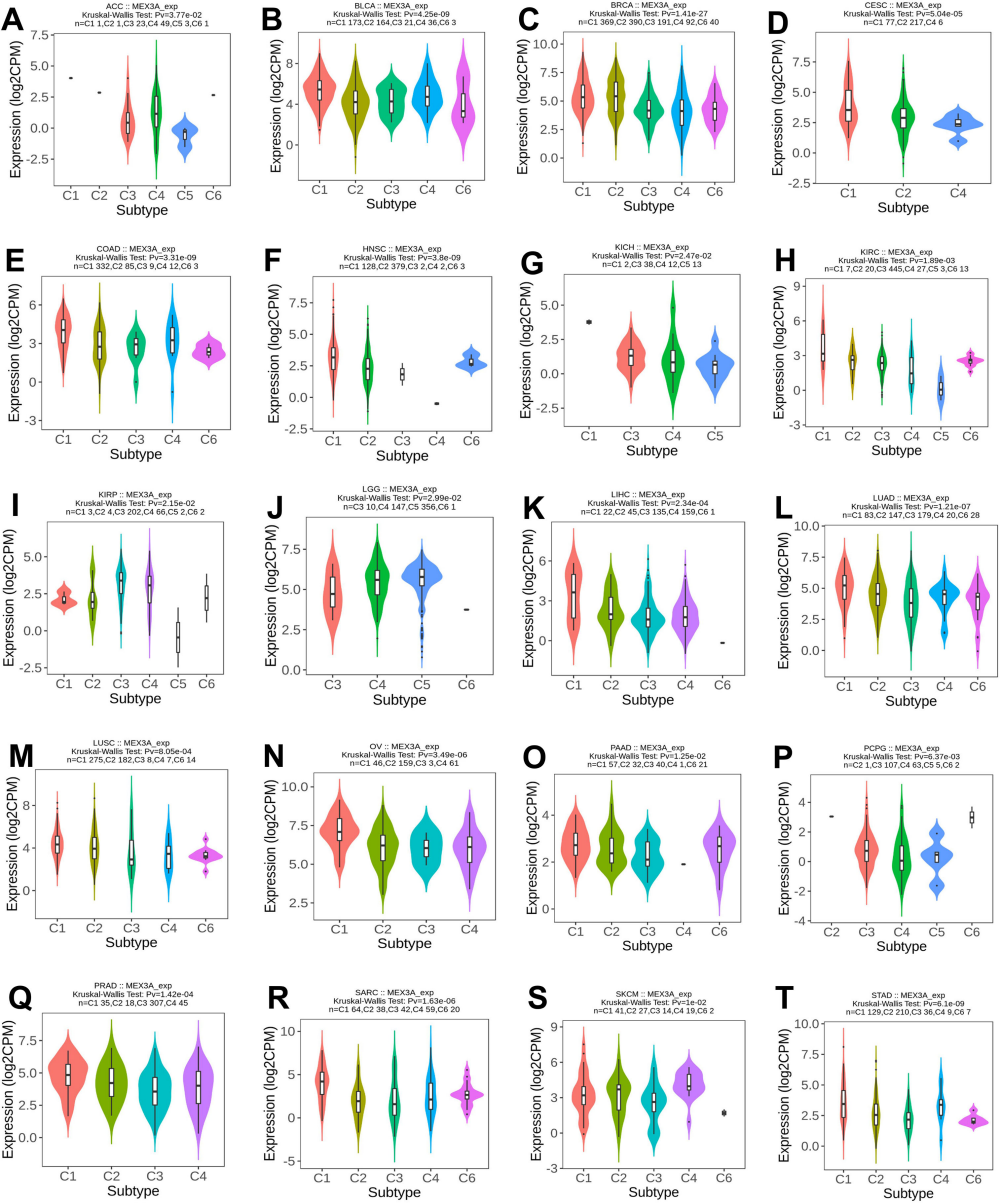
Pan-cancer analysis of MEX3A expression heterogeneity across molecular subtypes. MEX3A expression across six immune subtypes (C1–C6) in multiple cancer types **(A–T)**.

We systematically analyzed the distribution of MEX3A across established immune and molecular subtypes in multiple cancer types to further elucidate the immunological and molecular heterogeneity linked to its expression (**Figure 3**; **Supplementary Figure S2**). As shown in immune subtype analyses (**Figures 3A–T**), MEX3A expression differed significantly among the six immune subtypes (C1–C6) in a broad range of malignancies. Notably, higher MEX3A expression was frequently enriched in wound-healing (C1) and TGF-β–dominant (C6) subtypes, whereas relatively lower expression was observed in inflammatory (C3) or lymphocyte-depleted (C4) subtypes, although the specific patterns varied across cancer types. These findings suggest that MEX3A expression is closely linked to distinct immune phenotypes within the tumor microenvironment.

In addition, MEX3A expression exhibited significant heterogeneity across molecular subtypes defined by transcriptomic, epigenetic, or genomic features (**Supplementary Figures S2A–L**). In BRCA, MEX3A expression varied markedly among Basal, HER2-enriched, Luminal A, and Luminal B subtypes. Similar subtype-dependent expression patterns were observed in other cancers, including CRC, Glioblastoma multiforme (GBM), Head and neck squamous cell carcinoma (HNSC), Lung adenocarcinoma (LUAD), Ovarian cancer (OV), and Kidney renal papillary cell carcinoma (KIRP). Collectively, these results indicate that MEX3A expression is strongly associated with both immune and molecular stratification of tumors, underscoring its potential role in tumor heterogeneity and subtype-specific biology.

### 3.5 Correlation between MEX3A expression and immune-related molecules across cancers

**Figure 4:**
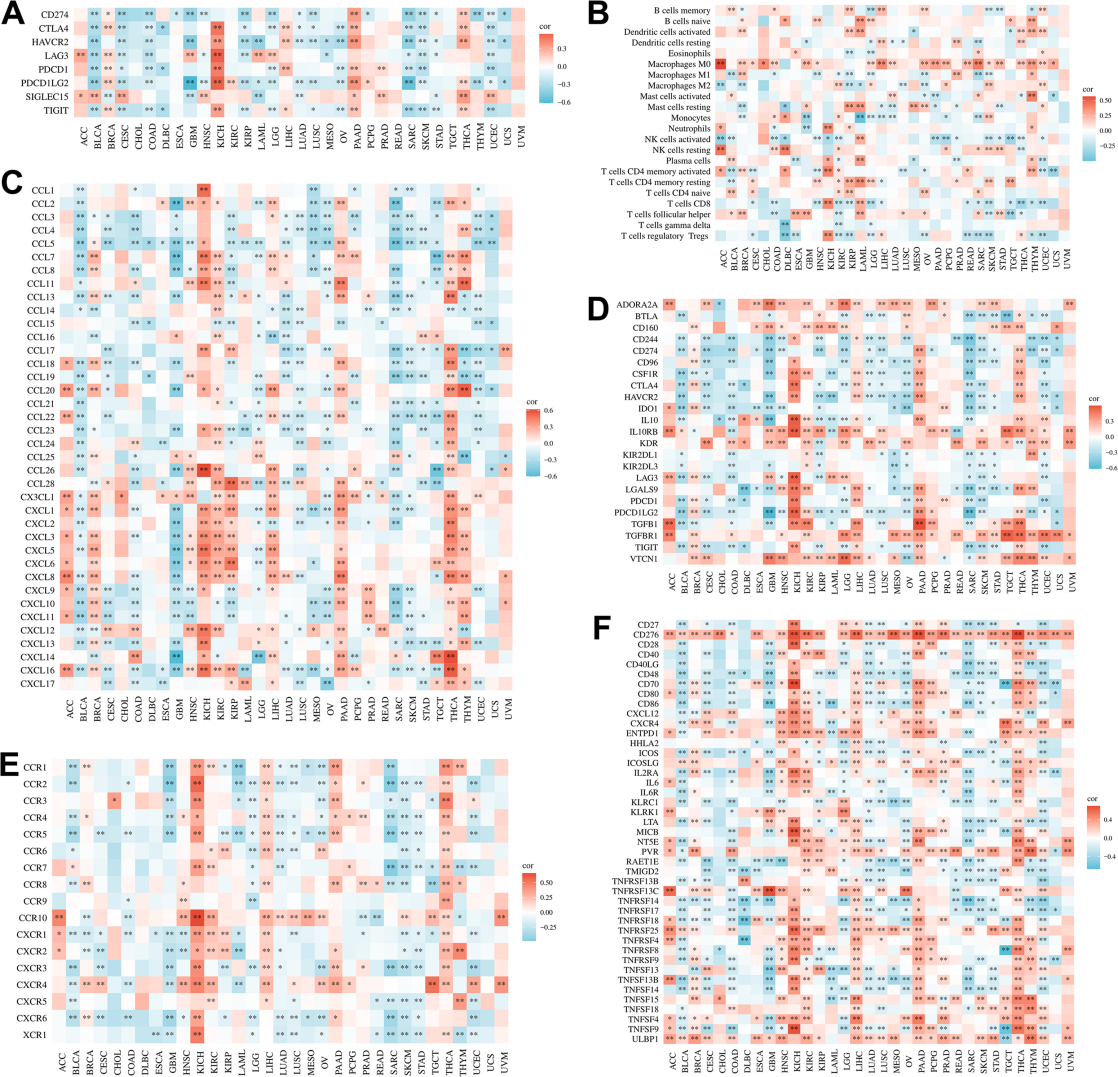
Pan-cancer correlation analyses of immune-related genes, chemokines, and receptors across human cancers. **(A)** Immune checkpoint genes. **(B)** Immune cell infiltration. **(C)** Chemokines. **(D)** Immunomodulatory genes. **(E)** Chemokine receptors. **(F)** Antigen presentation and co-stimulatory molecules. *p<0.05, **p<0.01, ***p<0.001.

We performed comprehensive correlation analyses between MEX3A expression and multiple immune-related features across pan-cancer cohorts to explore its immunological relevance. (**Figure 4**). As shown in **Figure 4A**, MEX3A expression exhibited significant correlations with classical immune checkpoint molecules, including PDCD1, CD274 (PD-L1), CTLA4, LAG3, TIGIT, and HAVCR2, in multiple tumor types, suggesting a potential link between MEX3A and immune checkpoint regulation. Consistently, **Figure 4B** demonstrated that MEX3A expression was closely associated with the infiltration levels of diverse immune cell populations, particularly T cells, macrophages, dendritic cells, NK cells, and B cells, although the direction and magnitude of these associations varied across cancer types, reflecting tumor-specific immune contexts. Chemokine-related analyses revealed that MEX3A expression was broadly correlated with numerous CCL and CXCL family members (**Figure 4C**), implying a possible role in modulating immune cell recruitment and trafficking within the tumor microenvironment. In addition, significant associations were observed between MEX3A and a wide panel of immunomodulatory genes, including immune inhibitory and stimulatory molecules (**Figure 4D**), further supporting its involvement in immune regulation. Receptor-level analyses showed that MEX3A expression was significantly correlated with multiple chemokine receptors (**Figure 4E**), reinforcing its potential influence on immune signaling networks. Finally, **Figure 4F** illustrated strong correlations between MEX3A and genes involved in antigen presentation, co-stimulation, and TNF superfamily signaling, highlighting a broad immunological footprint of MEX3A across cancers. Collectively, these findings indicate that MEX3A is extensively involved in shaping the tumor immune microenvironment in a cancer-type–dependent manner, supporting its potential value as an immune-related biomarker and therapeutic target.

### 3.6 Association of MEX3A with immune landscape, genomic instability, and mutation features in chRCC

**Figure 5:**
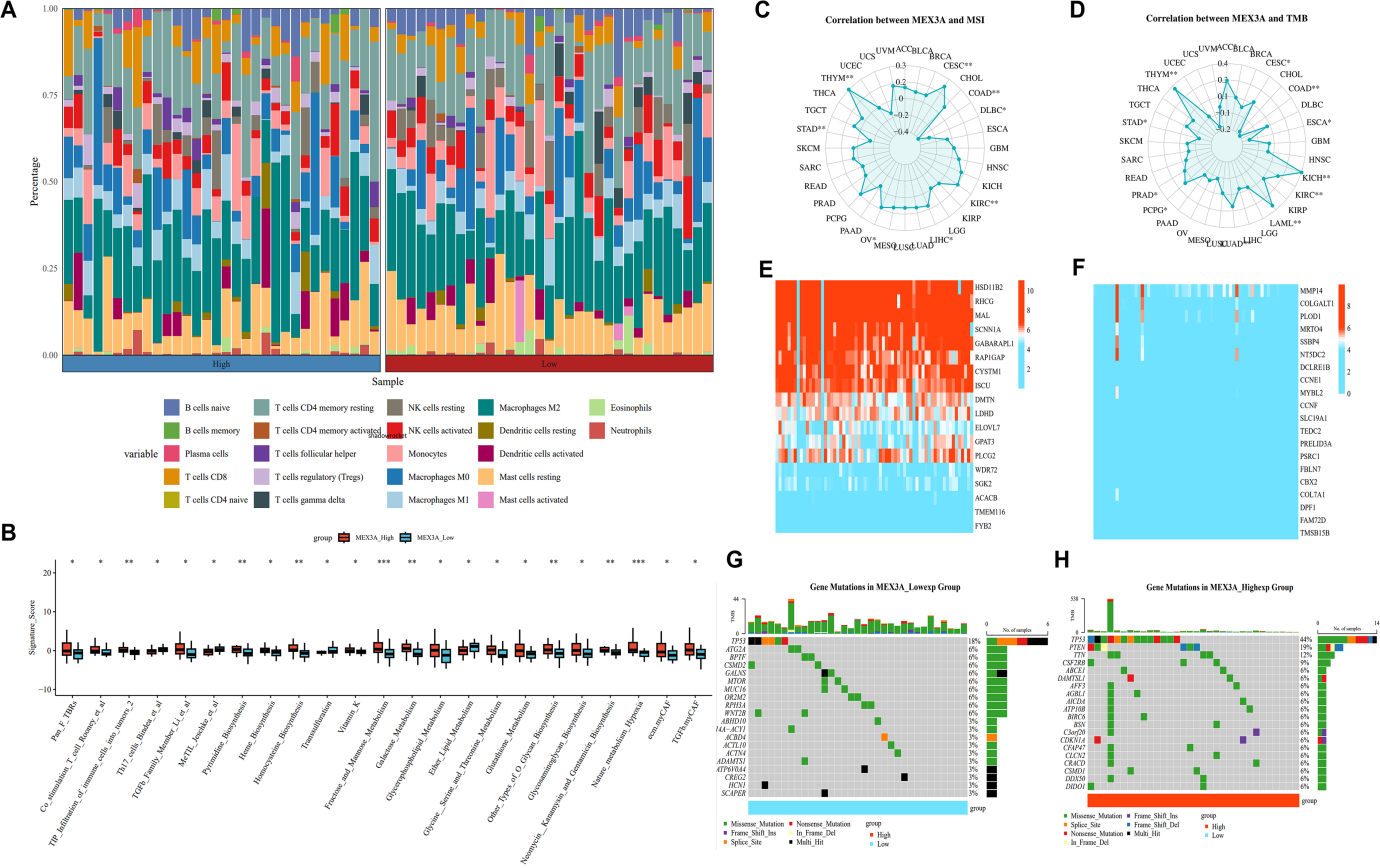
MEX3A expression correlates with immune microenvironment, genomic instability, and mutational landscapes in chRCC. **(A)** CIBERSORT analysis of immune cell infiltration differences between high and low MEX3A expression groups across cancer samples. **(B)** Immune microenvironment-related score comparisons between MEX3A high and low expression groups. **(C, D)** Correlation analysis between MEX3A expression and MSI and TMB. **(E)** Heatmap displays genes negatively correlated with MEX3A expression in chRCC. **(F)** Heatmap of genes positively correlated with MEX3A expression in chRCC. **(G)** Oncoplot of gene mutations in MEX3A low-expression group in chRCC. **(H)** Oncoplot of gene mutations in MEX3A high-expression group in chRCC. (*p < 0.05, **p < 0.01, ***p < 0.001).

We comprehensively analyzed the associations of MEX3A with immune cell infiltration, immune-related pathways, genomic instability indicators, transcriptomic alterations and somatic mutation profiles, thereby elucidating its biological implications in chRCC (**Figure 5**). As shown in **Figure 5A**, the immune cell composition differed markedly between chRCC samples with high and low MEX3A expression. Notable differences were observed in multiple immune cell subsets, including macrophage populations and T lymphocyte subsets, indicating that MEX3A expression is closely associated with remodeling of the tumor immune microenvironment in chRCC. Consistently, **Figure 5B** demonstrated significant alterations in multiple immune-related pathway signature scores between the two groups, suggesting that elevated MEX3A expression may influence immune activation, immune suppression, and inflammatory signaling pathways in chRCC. We next investigated the relationship between MEX3A expression and genomic instability. **Figures 5C, D** revealed a significant association between MEX3A expression and TMB, suggesting that MEX3A may be linked to underlying genomic alteration patterns in chRCC. At the transcriptomic level, differential expression analysis identified distinct gene expression signatures associated with MEX3A expression status. **Figure 5E** highlights genes preferentially upregulated in the MEX3A-high group, whereas **Figure 5F** displays genes enriched in the MEX3A-low group, indicating divergent transcriptional programs related to MEX3A expression in chRCC. Finally, somatic mutation analysis revealed clear differences in mutational landscapes between the two groups. As shown in **Figure 5G** and **Figure 5H**, chRCC tumors with low and high MEX3A expression exhibited distinct mutation frequencies and mutation types in key genes, reflecting genomic heterogeneity associated with MEX3A stratification. Collectively, these findings demonstrate that MEX3A expression in chRCC is closely associated with immune infiltration patterns, immune-related functional pathways, genomic instability metrics, transcriptional alterations, and somatic mutation characteristics, underscoring its potential role in shaping the tumor microenvironment and genomic landscape of chRCC.

### 3.7 Drug sensitivity and drug–response correlation analysis of MEX3A in chRCC

**Figure 6:**
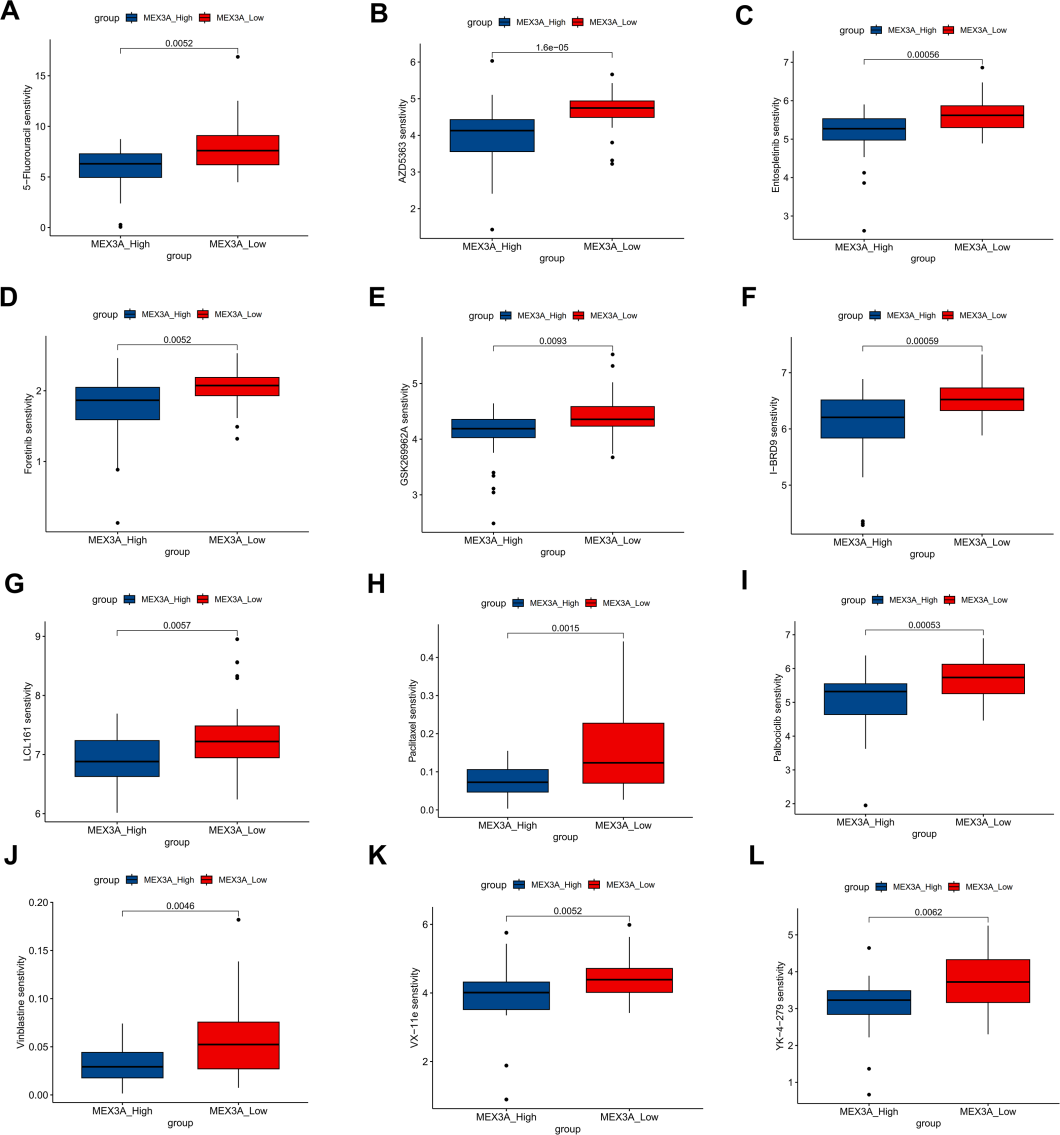
Association between MEX3A expression and predicted IC50 values of anticancer drugs in chRCC. chRCC samples were stratified into high- and low-MEX3A expression groups based on the median MEX3A level. Boxplots illustrate the predicted drug sensitivity (IC50 values) of multiple anticancer agents between the two groups, including 5-fluorouracil **(A)**, AZD5363 **(B)**, etoposide **(C)**, foretinib **(D)**, GSK269962A **(E)**, I-BRD9 **(F)**, LCL161 **(G)**, paclitaxel **(H)**, palbociclib **(I)**, vinblastine **(J)**, VX-11e **(K)**, and YK-4-279 **(L)**.

Given the prognostic value of MEX3A in chRCC, we next performed in silico drug sensitivity prediction to investigate whether MEX3A expression is associated with therapeutic response to commonly used anti-cancer agents, aiming to explore its potential as a predictive biomarker for treatment in chRCC (**Figures 6A–L**; **Supplementary Figure S3**). Tumors with high MEX3A expression exhibited significantly higher predicted IC50 values for multiple chemotherapeutic and targeted agents, including 5-fluorouracil, AZD5363, entospletinib, foretinib, GSK269962A, l-BRD9, LCL161, paclitaxel, palbociclib, vinblastine, VX-11e, and YK-4-279 (all *p* < 0.01). Consistently, correlation analyses demonstrated that MEX3A expression was negatively correlated with drug sensitivity, as reflected by positive associations with predicted IC50 values. These results indicate that elevated MEX3A expression is associated with reduced responsiveness to anticancer therapies in chRCC.

### 3.8 Functional effects of MEX3A knockdown in renal cancer cells

**Figure 7:**
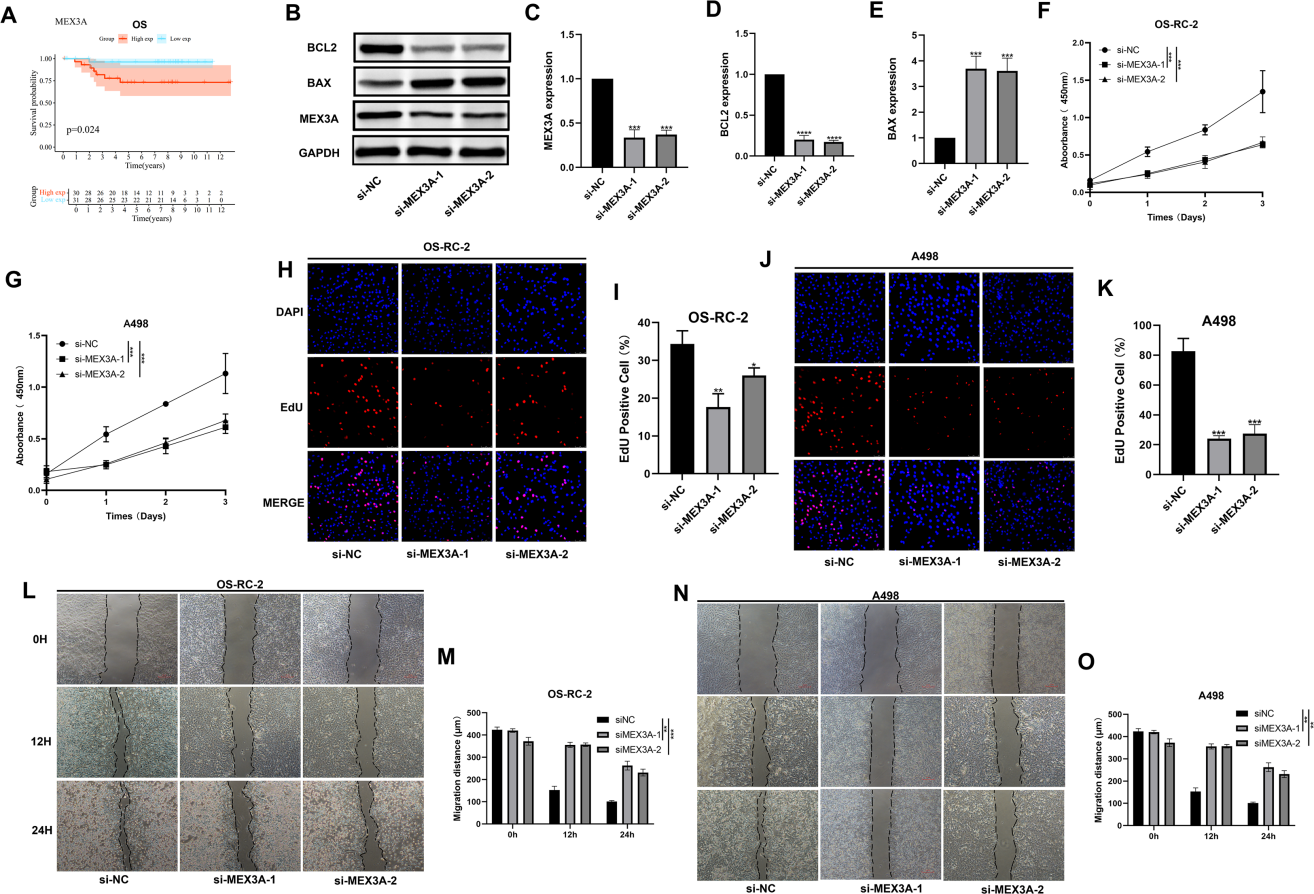
MEX3A promotes malignant phenotypes in renal cancer cells. **(A)** Kaplan-Meier survival analysis of overall survival (OS) in chRCC patients stratified by MEX3A expression. **(B)** Western blot analysis of MEX3A, BCL2, and BAX protein levels in OS-RC-2 cells transfected with si-NC, si-MEX3A-1, or si-MEX3A-2. **(C-E)** Quantitative analysis of MEX3A, BCL2, and BAX expression normalized to GAPDH. **(F, G)** CCK-8 assays were performed to assess cell viability in OS-RC-2 **(F)** and A498 **(G)** cells after MEX3A knockdown. **(H-K)** EdU staining was conducted to evaluate cell proliferation in OS-RC-2 **(H, I)** and A498 **(J, K)** cells, with quantification of EdU-positive cells. **(L-O)** Wound-healing assays were used to detect migratory ability in OS-RC-2 **(L, M)** and A498 **(N, O)** cells, with measurement of migration distance at 0, 12, and 24 h. Data are presented as the mean ± SD of three independent experiments. (*p < 0.05, **p < 0.01, ***p < 0.001 vs. si-NC).

Functional experiments validating the role of MEX3A are presented in **Figure 7**. Consistent with our pan-cancer prognostic findings, Kaplan-Meier survival analysis confirmed that high MEX3A expression was significantly associated with reduced OS in chRCC patients (**Figure 7A**). To further validate the oncogenic role of MEX3A predicted by bioinformatics analysis, we performed loss-of-function experiments in two renal cancer cell lines, OS-RC-2 and A498. First, we confirmed the knockdown efficiency of two independent siRNAs targeting MEX3A (si-MEX3A-1 and si-MEX3A-2) via Western blot (**Figures 7B, C**). As shown in **Figures 7B–E**, MEX3A silencing significantly downregulated the expression of the anti-apoptotic protein BCL2 and upregulated the pro-apoptotic protein BAX, indicating that MEX3A may regulate apoptosis in renal cancer cells.

Next, CCK-8 assays demonstrated that knockdown of MEX3A markedly suppressed the viability of both OS-RC-2 and A498 cells in a time-dependent manner (**Figures 7F, G**). Consistently, EdU staining revealed a significant reduction in the number of EdU-positive proliferating cells in the si-MEX3A groups compared with the si-NC control group (**Figures 7H–K**). Furthermore, wound-healing assays showed that MEX3A knockdown significantly impaired the migratory ability of OS-RC-2 and A498 cells, with a marked decrease in wound closure at 12 h and 24 h post-scratching (**Figures 7L–O**). Collectively, these *in vitro* results strongly support the oncogenic function of MEX3A in promoting proliferation, migration, and inhibiting apoptosis in renal cancer cells, which is consistent with its poor prognostic value in chRCC.

## 4 Discussion

In the present study, we performed a comprehensive pan-cancer and integrative analysis to elucidate the biological and clinical relevance of MEX3A, with a particular emphasis on chRCC. By leveraging large-scale multi-omics datasets and experimental validation, we demonstrate that MEX3A is aberrantly upregulated in multiple malignancies and exerts a cancer-type–dependent prognostic effect (21). Our pan-cancer survival analyses revealed that elevated MEX3A expression was associated with unfavorable clinical outcomes in several tumor types, including ACC, LIHC, SARC, MESO, and chRCC, whereas weaker or nonsignificant associations were observed in other cancers. These findings highlight the context-specific nature of MEX3A-mediated oncogenic activity, underscoring that its biological functions are shaped by tumor lineage, molecular background, and microenvironmental context. Notably, chRCC emerged as one of the tumor types in which MEX3A showed consistent prognostic relevance across multiple survival endpoints, suggesting a particularly important role in this rare renal carcinoma subtype. Given the limited availability of prognostic biomarkers for chRCC, our findings provide novel insights into risk stratification and disease progression in this understudied malignancy. Notably, chRCC is a rare subtype with limited therapeutic strategies and few effective molecular markers. Our study identified MEX3A as a novel prognostic factor and oncogenic driver in chRCC, filling the current research gap.

Beyond prognostic associations, our study reveals that MEX3A is closely linked to immune regulation and genomic instability, especially in chRCC. Immune infiltration analyses demonstrated that MEX3A expression stratified chRCC tumors into distinct immune phenotypes, characterized by differential infiltration of macrophage subsets and T lymphocytes, as well as altered immune-related pathway activities. These observations suggest that MEX3A may contribute to remodeling of the tumor immune microenvironment, potentially influencing immune surveillance and tumor–immune interactions (22, 23). Consistently, MEX3A expression was significantly correlated with immune checkpoint molecules and immunomodulatory genes across cancers, implying a broader role in immune evasion mechanisms. In parallel, we identified significant associations between MEX3A expression and genomic instability indicators, including tumor mutation burden and microsatellite instability. Together with the distinct mutational landscapes observed between MEX3A-high and -low chRCC tumors, these results suggest that MEX3A may be involved in maintaining genomic states that favor tumor adaptation and progression. Given that both immune contexture and genomic instability are critical determinants of therapeutic response, the multifaceted associations of MEX3A position it as a potential integrative biomarker linking tumor genomics, immunity, and clinical behavior.

Consistent with its diverse roles reported in multiple malignancies, our pan-cancer analysis revealed a significant prognostic association between MEX3A and patient survival. In line with previous studies, MEX3A has been shown to promote cancer progression through multiple mechanisms, including the regulation of RNA metabolism, activation of oncogenic signaling, and modulation of immune-related pathways (24). We further elaborate on the potential link between MEX3A and immune regulation as well as therapeutic resistance, providing a broader context for our findings in chRCC. Importantly, our findings extend beyond descriptive associations by providing evidence for the therapeutic relevance of MEX3A in chRCC. Drug sensitivity analyses revealed that high MEX3A expression was consistently associated with reduced sensitivity to a broad panel of chemotherapeutic and targeted agents, including DNA-damaging drugs, cell cycle inhibitors, and pathway-specific inhibitors. Correlation analyses further confirmed significant negative relationships between MEX3A expression and drug responsiveness, suggesting that MEX3A may contribute to intrinsic or acquired drug resistance. These computational predictions were supported by functional experiments, in which MEX3A knockdown significantly suppressed proliferation and migration of renal cancer cells and altered the expression of apoptosis-related proteins. Collectively, these results indicate that MEX3A not only serves as a prognostic marker but also actively promotes malignant phenotypes and therapeutic resistance in chRCC. From a translational perspective, MEX3A may therefore represent a promising candidate for patient stratification and a potential target to enhance treatment efficacy. In line with its functional relevance in tumor metabolism and proliferation, our study further highlights the potential oncogenic role of MEX3A in chRCC, supporting the broader implication of MEX3A as a conserved regulator across different tumor types. Nevertheless, several limitations should be acknowledged. The reliance on retrospective public datasets may introduce selection bias, and the mechanistic underpinnings of MEX3A-mediated immune modulation and drug resistance remain to be fully elucidated. Future studies integrating *in vivo* models, single-cell analyses, and prospective clinical validation will be essential to further clarify the functional roles of MEX3A and to evaluate its potential as a therapeutic target. Despite these limitations, our integrative analysis provides a comprehensive framework for understanding the multifaceted role of MEX3A in cancer and highlights its particular significance in chRCC.

## 5 Conclusion

In summary, this study systematically characterized the role of MEX3A across cancers and highlighted its clinical relevance in chRCC. Elevated MEX3A expression was associated with unfavorable prognosis and distinct alterations in the tumor immune microenvironment and genomic features in chRCC. Moreover, high MEX3A levels were consistently linked to reduced sensitivity to multiple chemotherapeutic and targeted agents, suggesting a potential role in therapeutic resistance. Functional analyses in renal cancer cells further supported the oncogenic properties of MEX3A in promoting tumor progression. Collectively, these findings indicate that MEX3A may serve as a valuable prognostic biomarker and a promising predictor of treatment response in chRCC, providing a rationale for future studies exploring MEX3A as a potential therapeutic target.

## Data Availability

The datasets presented in this study can be found in online repositories. The names of the repository/repositories and accession number(s) can be found below: https://portal.gdc.cancer.gov, TCGA database http://cbioportal.org, cBIOPORTAL database https://www.proteinatlas.org, HPA database https://registry.opendata.aws/ccle, CCLE database http://gepia2.cancer-pku.cn, GEPIA2 database.

## Supplementary material

Supplementary Figure 1 Tissue - specific expression profiles of a MEX3A across human organs. A. MEX3A gene expression at RNA and protein levels across major human organ systems. B. RNA expression of MEX3A in various human tissues. C. RNA expression of MEX3A across human tissues from the GTEx dataset. D. MEX3A gene expression across multiple samples for each tissue type, providing insights into expression variability.

Supplementary Figure 2 MEX3A expression profiling across immune subtypes of diverse cancers. MEX3A expression across distinct immune subtypes for various cancer types (A-L).

Supplementary Figure 3 Correlation analysis of MEX3A expression with drug sensitivity in chRCC. (A-L) illustrate the correlation between MEX3A expression and the sensitivity to different drugs in chRCC.
