# Supplementary figures and images for: Pan-cancer analysis reveals the prognostic and therapeutic relevance of MEX3A with a focus on chromophobe renal cell carcinoma
Source: Front Oncol. 2026 Apr 29;16:1802679. doi: 10.3389/fonc.2026.1802679 (PMC13167595; doi:10.3389/fonc.2026.1802679)

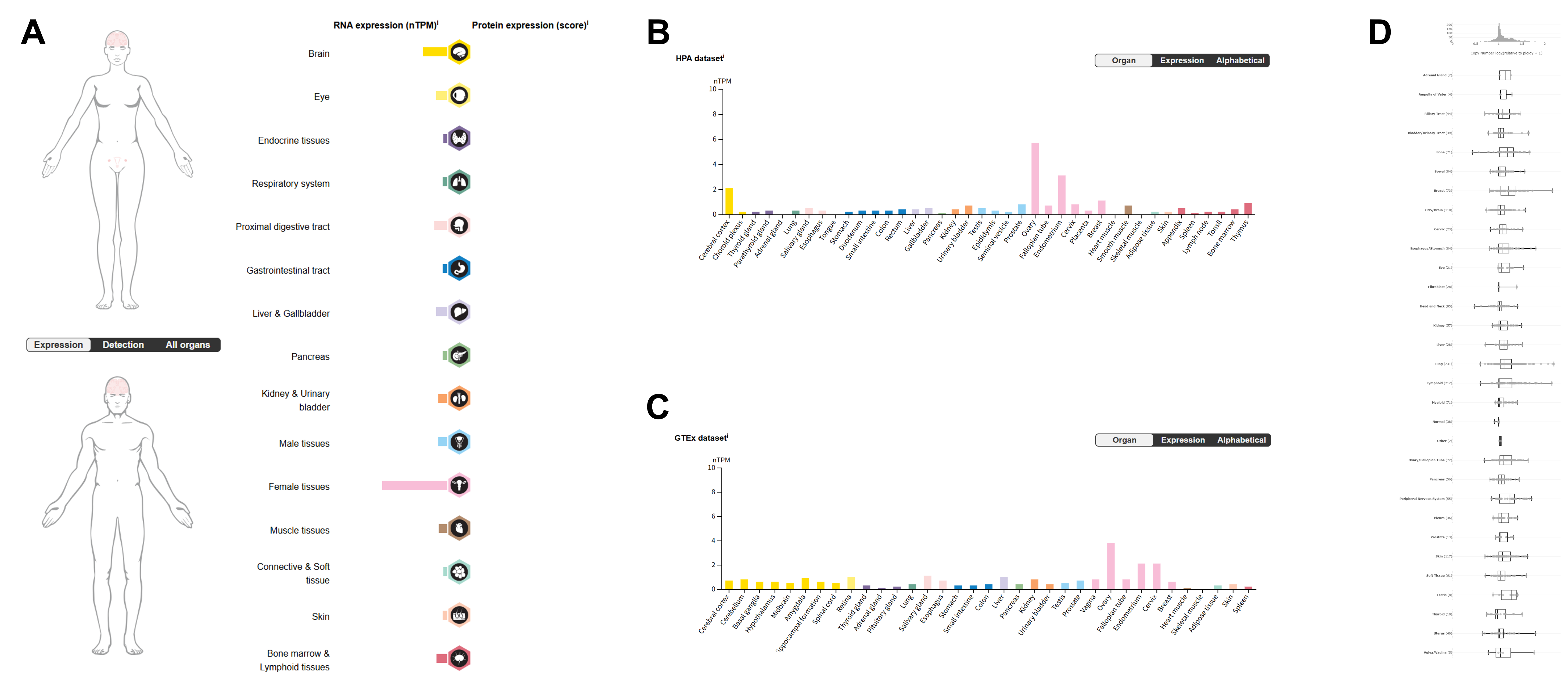

Supplement: Supplementary Figure 1 — Tissue - specific expression profiles of a MEX3A across human organs. A. MEX3A gene expression at RNA and protein levels across major human organ systems. B. RNA expression of MEX3A in various human tissues. C. RNA expression of MEX3A across human tissues from the GTEx dataset. D. MEX3A gene expression across multiple samples for each tissue type, providing insights into expression variability. [file Image1.tif]

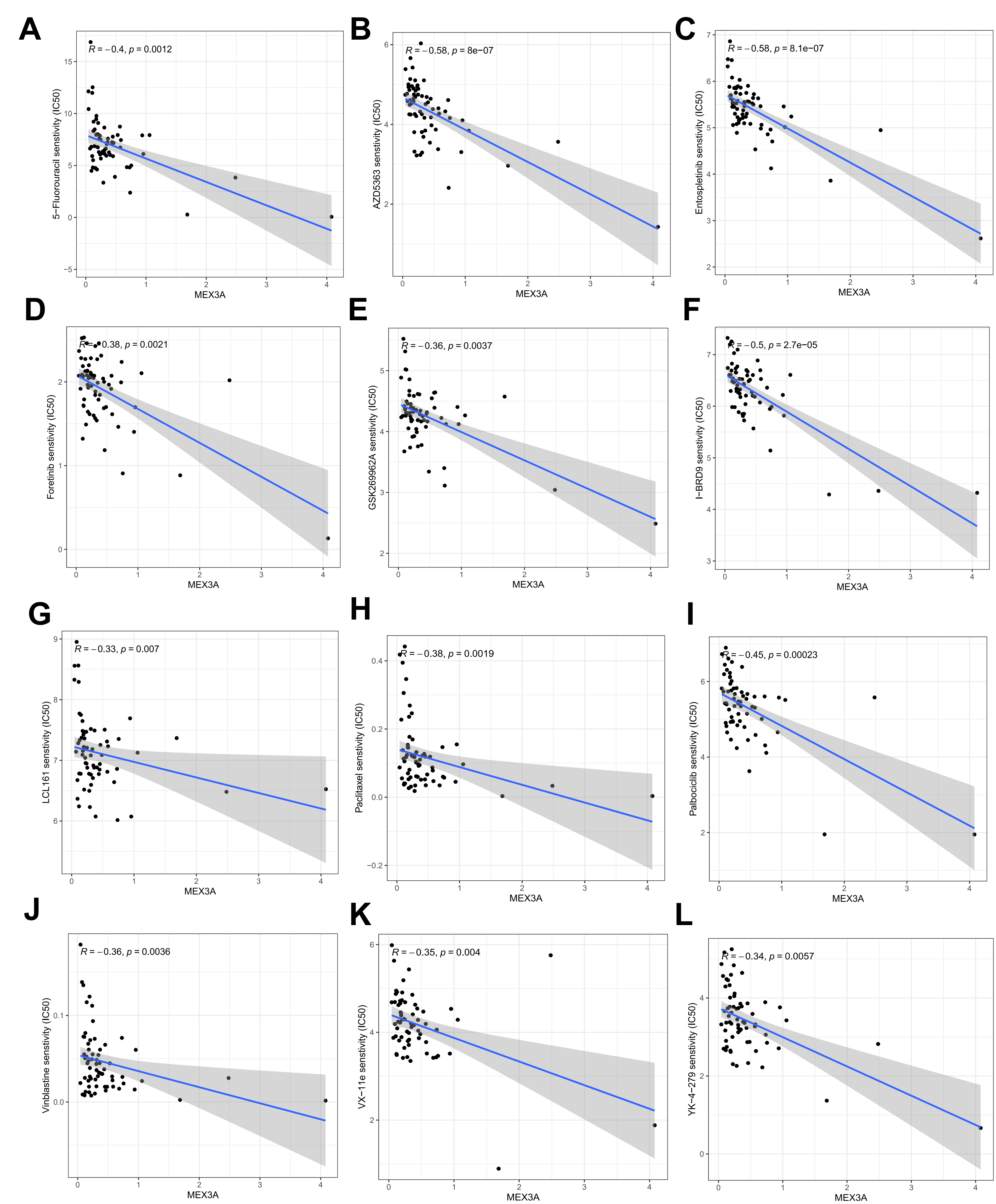

Supplement: Supplementary Figure 3 — Correlation analysis of MEX3A expression with drug sensitivity in chRCC. (A-L) illustrate the correlation between MEX3A expression and the sensitivity to different drugs in chRCC. [file Image3.tif]
