# Supplementary material for: Pan-cancer analysis reveals the prognostic and therapeutic relevance of MEX3A with a focus on chromophobe renal cell carcinoma
Source: Front Oncol. 2026 Apr 29;16:1802679. doi: 10.3389/fonc.2026.1802679 (PMC13167595; doi:10.3389/fonc.2026.1802679)
Supplement: Supplementary file 4 [file Table1.docx]

| siRNA Name | Sense Strand (5′→3′) | Antisense Strand (5′→3′) |
| --- | --- | --- |
| si-MEX3A-NC | UUCUCUCUCCGAACGUGUCUCUCUGACGU | ACGUUCAGAGAGACACGUUCGGAGAGAGAA |
| si-MEX3A-1 | GCAAGAUCCUCGAGUACAA | UUGUACUCGAGGAUCUUGC |
| si-MEX3A-2 | AGGCAAGGCTGCAAGATTAA | CUUAUCUUGCAGCCUUGCCU |

*Table1 si-MEX3A related sequences
